# Künstliche Intelligenz im urologischen Versorgungsalltag: Ergebnisse des KI-Barometers

**DOI:** 10.1007/s00120-026-02852-1

**Published:** 2026-05-19

**Authors:** Nicolas Carl, Frederik Wessels, Jonathan Jeutner, Sebastian Frees, Mike Wenzel, Felix Chun, Julian P. Struck, Hendrik Borgmann, Severin Rodler

**Affiliations:** 1 Klinik für Urologie und Urochirurgie, Universitätsklinik Heidelberg Campus Mannheim, Theodor-Kutzer Ufer 1–3, 68167 Mannheim, Deutschland; 2 Klinik für Urologie, Charité – Universitätsmedizin Berlin, Berlin, Deutschland; 3 Urologische Gemeinschaftspraxis, Waldstraße 6, Mainz-Gonsenheim, Deutschland; 4 Klinik für Urologie, Universitätsklinikum Frankfurt am Main, Frankfurt am Main, Deutschland; 5 Klinik für Urologie und Kinderurologie, Universitätsklinikum Brandenburg an der Havel, Brandenburg an der Havel, Deutschland; 6 Klinik für Urologie, Universitätsklinikum Schleswig-Holstein Campus Kiel, Kiel, Deutschland; 7 Arbeitskreis für Künstliche Intelligenz und Digitalisierung, Deutsche Gesellschaft für Urologie e.V., 40474 Uerdinger Str. 64, Düsseldorf, Deutschland

**Keywords:** Künstliche Intelligenz, Versorgungslage, KI Nutzung, Anwendungsfälle, Bedarfsanalyse, Artificial intelligence, Health care services, Artificial intelligence use, Application examples, Needs assessment

## Abstract

**Hintergrund:**

Die urologische Versorgung in Deutschland ist im Wandel und neue Anwendungen mit künstlicher Intelligenz (KI) bieten das Potential, Prozesse zu automatisieren und Personal zu entlasten. Ziel war es daher, die Arbeitsbelastung, aktuelle KI-Nutzung sowie Vertrauen und Erwartungen im Kontext der aktuellen Versorgungslage systematisch zu beschreiben.

**Methoden:**

Deutschlandweite, anonyme Querschnittbefragung im urologischen Versorgungsbereich von Januar bis April 2026. Die Auswertung erfolgte explorativ und deskriptiv.

**Ergebnisse:**

Insgesamt nahmen 433 Personen teil, überwiegend Ärzt:innen. 79 % berichteten über ein gestiegenes Patientenaufkommen, 69 % gaben an, dass die verfügbare Zeit für eine qualitativ hochwertige Versorgung nicht ausreiche. Als Haupttreiber des Zeitdrucks wurde administrativer und dokumentationsbezogener Aufwand genannt (93,1 %). Allgemein zugängliche KI-Tools werden deutlich häufiger genutzt, 74,6 % verwenden KI privat und/oder beruflich, während medizinisch zugelassene Systeme nur von einer Minderheit eingesetzt werden. Die Nutzung konzentriert sich auf informations- und textbasierte Aufgaben wie Recherche, Übersetzung und Textentwürfe. Gleichzeitig bestehen Hinweise für einen hohen, bislang ungedeckter Bedarf, insbesondere für Kodierung, Dokumentation, Bildanalyse und Medikationsmanagement. Angegebene Voraussetzungen für Vertrauen in KI sind überprüfbare Qualität (82,4 %), evidenzbasierte Datenquellen (72,1 %), klare regulatorische Rahmenbedingungen (70,0 %) und Integration in IT-Strukturen (63,7 %). Siebenundfünfzig Prozent der Teilnehmenden erwarten künftig eine Zeitersparnis durch KI.

**Fazit:**

Die urologische Versorgung wird von den Befragten als zunehmend verdichtet wahrgenommen. KI wird bereits breit genutzt, derzeit jedoch v. a. in Form allgemein zugänglicher Anwendungen. Die Ergebnisse deuten zudem auf einen bislang nicht gedeckten Bedarf an KI-Anwendungen für ausgewählte kleinteilige Alltagsaufgaben hin, insbesondere sofern Anforderungen an Qualität, Transparenz und Governance erfüllt sind.

**Graphic abstract:**

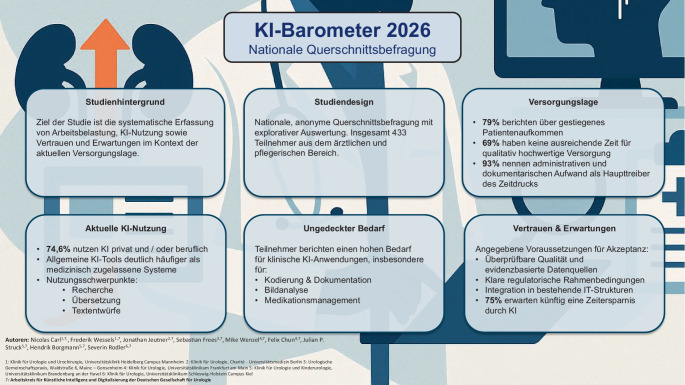

## Abstract (en)

**Background:**

Urological care in Germany is undergoing transformation, while novel technologies with artificial intelligence (AI) have the potential to automate processes and support caregivers. The aim was therefore to systematically describe workload, current AI use, as well as trust and expectations within the context of the current healthcare landscape.

**Methods:**

Nationwide anonymous cross-sectional survey conducted from January to April 2026 among professionals in urological care. Analysis was exploratory and descriptive.

**Results:**

A total of 433 participants responded, predominantly physicians. Increased patient volume was reported by 79%, and 69% stated that available time was insufficient for high-quality care. Administrative and documentation-related burden was identified as the main driver of time pressure by 93,1%. General-purpose AI tools were used substantially more often than clinic-specific applications. While 74,6% reported both private and/or professional AI use, certified medical AI applications were rarely used. Current use focused on information- and text-based tasks such as information retrieval, translation, and text drafting. At the same time, substantial unmet demand was observed, particularly for coding, documentation, imaging analysis, and medication management. Key prerequisites included verifiable output quality (82,4%), evidence-based data sources (72,1%), clear regulatory frameworks (70,0%), and integration into information infrastructures (63,7%). In all, 75,1% expected future time savings through AI.

**Conclusion:**

Urological care is perceived by respondents as increasingly intensified. AI is already widely used, but currently mainly in the form of general-purpose applications. The findings also indicate an unmet need for AI applications for selected small-scale routine tasks, particularly if requirements for quality, transparency, and governance are met.

## Einleitung

Die urologische Versorgung in Deutschland steht unter dem kombinierten Druck des demografischen Wandels, des Fachkräftemangels und des strukturellen Umbaus der Krankenhausreform. Der Anteil der Bevölkerung über 67 Jahre wird bis 2035 auf etwa 25 % steigen, wodurch Versorgungsbedarf und Morbiditätslast zunehmen [1]. Gleichzeitig verschärfen sich Personalengpässe bei einer erwarteten Lücke von 280.000–690.000 Pflegekräften im Jahr 2049 [2]. Auf der stationären Seite ist zudem ein Konzentrationsprozess sichtbar. Seit 1991 sinkt die Zahl der Krankenhäuser von 2411 auf 1841 im Jahr 2024, während Insolvenzen auf eine anhaltende wirtschaftliche Instabilität der verbleibenden Krankenhäuser hinweisen [3]. Dies trägt zur Verdichtung klinischer Arbeitsabläufe bei, was sich in Studien beispielhaft darstellen lässt. An einer Universitätsklinik stehen Ärzt:innen durchschnittlich nur etwa 4 min für die direkte Kommunikation mit Patient:innen sowie rund 20 s für Gespräche mit Angehörigen zur Verfügung [4]. Der *Clinician of the Future Report *2025 zeigt zudem, dass befragte Ärzt:innen unter Zeitdruck leiden [5]. Von über 2000 Befragten berichten 28 % über unzureichende Zeit für hochwertige Versorgung, 69 % behandeln mehr Patienten als vor 2 Jahren und es werden klare Erwartungen an künstliche Intelligenz (KI) als Entlastungsinstrument formuliert [5].

Durch das Potenzial, klinische Aufgaben zu unterstützen oder teilweise zu automatisieren, rücken KI-Anwendungen als Hebel zur Entlastung in den Fokus. KI-Systeme werden wissenschaftlich ausgiebig exploriert und zeigen eine ausgesprochene theoretische Leistungsfähigkeit in der Unterstützung der Arzt-Patienten-Kommunikation [6–8], der Analyse diagnostischer Bildgebung [9] sowie als autonome multimodale Entscheidungsunterstützung im Tumorboard [10–12]. Darüber hinaus verfügen große Sprachmodelle („large language models“, LLM) über die Fähigkeit, medizinische Texte semantisch zu analysieren, strukturiert auszuwerten und kontextbezogen zu verarbeiten. Beispielsweise können LLM Texte verfassen, Informationen aus Befunden sowie Arztbriefen extrahieren, medizinisches Fachwissen anwenden und vieles mehr [12–14].

Insgesamt weist die bisherige Evidenz auf ein breites Anwendungsspektrum und ein enormes Potenzial von KI zur Unterstützung klinischer Arbeitsprozesse hin. Jedoch ist die Implementierung im Versorgungsalltag begrenzt und überwiegend für Anwendungen in der Forschung dokumentiert [15]. Demgegenüber zeigt eine aktuelle Studie des TÜV-Verbands, dass generative KI (i.e. Chatbots) bereits breite Anwendung in der Allgemeinbevölkerung findet [16].

Vor diesem Hintergrund verfolgt die vorliegende Studie das Ziel, ein aktuelles Meinungsbild zum urologischen Versorgungsalltag sowie zum gegenwärtigen und zukünftig erwarteten Einsatz von KI zu erheben. Erhoben wurden die wahrgenommene Arbeitsbelastung, Ursachen für Zeitdruck sowie Nutzung, Vertrauen und Erwartungen gegenüber KI.

## Material und Methoden

### Studiendesign und Datenerhebung

Das KI-Barometer wurde als Querschnittstudie angelegt. Die Datenerhebung erfolgte im Zeitraum zwischen Januar und April 2026. Eingeladen wurden medizinisch tätige Mitarbeitende im urologischen Versorgungsbereich per E‑Mail. Die Teilnahme war freiwillig.

### Datenschutz und Ethik

Die Befragung erfolgte anonym. Es wurden keine personenbezogenen oder rückverfolgbaren Daten erhoben. Die Angabe demografischer Merkmale war freiwillig. Die Befragung erfolgte mit positivem Votum der Ethikkommission II der Universität Heidelberg (ID: *2026-520*).

### Fragebogenerstellung

Der Fragebogen orientierte sich thematisch am *Clinician of the Future Report 2025* [5] sowie an der *ChatGPT*4*-Studie* *2025 *des TÜV-Verbands [16]. Die Items wurden eigenständig formuliert und auf den urologischen Versorgungskontext angepasst.

Der Fragebogen wurde in sechs thematische Module gegliedert:

1. Arbeitsbelastung, Zeitdruck und klinischer Alltag,
2. allgemeine Nutzung von KI-Tools,
3. aufgabenspezifische Nutzung von KI-Tools,
4. Vertrauen in KI-gestützte Systeme,
5. Erwartungen an zukünftige Auswirkungen von KI sowie
6. freiwillige demografische Angaben.

### Statistische Analyse

Die Auswertung erfolgte deskriptiv und explorativ. Da der Fragebogen nicht formal psychometrisch validiert und keine Skalenprüfung durchgeführt wurde, erfolgte die Auswertung der Likert-Items auf Ebene ordinaler Einzelitems ohne Skalenaggregation. Ergebnisse werden als absolute und relative Häufigkeiten dargestellt. Analysen erfolgten in R (Version 4.2.3; R Core Team, R Foundation for Statistical Computing, Wien, Österreich), Abbildungen wurden mit R und GraphPad Prism 10 (GraphPad Software, Boston, Massachusetts, USA) erstellt.

## Ergebnisse

### Stichprobe und beruflicher Hintergrund

Von ca. 5300 eingeladenen Personen nahmen 433 an der Befragung teil, was einer Rücklaufquote von ca. 8,2 % entspricht. Bezogen auf die gültigen Angaben zum beruflichen Hintergrund war der überwiegende Anteil der Teilnehmenden ärztlich tätig (85,1 %; *n* = 363), während 14,9 % (*n* = 63) angaben, im pflegerischen Bereich tätig zu sein. Der größte Anteil der Befragten arbeitete an einer Universitätsklinik (41,7 %; *n* = 178). Weitere 32,4 % (*n* = 138) waren im niedergelassenen Bereich tätig. 12,5 % (*n* = 53) arbeiteten in einem Krankenhaus der Maximalversorgung, während 11,1 % (*n* = 47) in Einrichtungen der Regel- und Schwerpunktversorgung beschäftigt waren.

### Arbeitsbelastung, Zeitdruck und klinischer Alltag

Die Mehrheit der Befragten berichtete eine zunehmende Arbeitsverdichtung im Versorgungsalltag. So stimmten 79 % der Aussage zu, heute mehr Patient:innen zu behandeln als noch vor 2 Jahren. Gleichzeitig gaben 78 % an, dass die zunehmende medizinische Komplexität der Patient:innen die verfügbare Zeit für deren Versorgung begrenze. Zudem berichteten 77 %, dass die im klinischen Alltag verfügbare Zeit nicht ausreiche, um komplexe medizinische Sachverhalte verständlich zu erklären.

Insgesamt empfanden es 68 % der Befragten als herausfordernd, mit neuen medizinischen und wissenschaftlichen Entwicklungen in der Urologie Schritt zu halten. Darüber hinaus gaben 52 % an, dass sich die interdisziplinäre Zusammenarbeit mit anderen Gesundheitsdienstleister:innen schwierig gestalte. Medizinische Fehlinformationen, beispielsweise aus dem Internet oder aus sozialen Medien, erschwerten aus Sicht von 51 % der Befragten die Versorgung von Patient:innen.

**Abb. 1:**
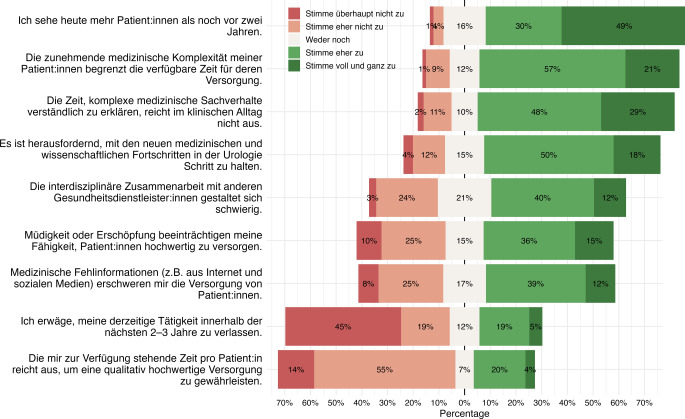
Likert-Plot zur Arbeitsbelastung im urologischen Versorgungsalltag (*n* = 433)

Hinsichtlich der verfügbaren Zeitressourcen verneinten 69 % der Befragten die Aussage, dass ihnen ausreichend Zeit pro Patient:in zur Verfügung stehe, um eine qualitativ hochwertige Versorgung zu gewährleisten. Zudem gaben 51 % an, dass Müdigkeit oder Erschöpfung ihre Fähigkeit zur Patient:innenversorgung beeinträchtige. Einen möglichen beruflichen Ausstieg thematisierten 24 % der Befragten, die angaben, ihre derzeitige Tätigkeit innerhalb der nächsten 2 bis 3 Jahre verlassen zu wollen; die Mehrheit von 69 % verneinte diese Aussage jedoch. Die Ergebnisse sind in Abb. 1 dargestellt.

**Abb. 2:**
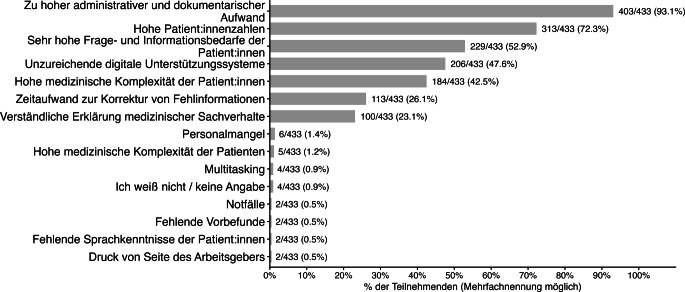
Hauptgründe für Zeitdruck (Mehrfachnennungen; *n* = 433)

Im Anschluss konnten die Teilnehmenden ihre Hauptursachen für Zeitdruckes nennen. Als häufigster Faktor wurde ein hoher administrativer und dokumentationsbezogener Aufwand genannt (93,1 %). Weitere Gründe waren hohe Patient:innenzahlen (72,3 %), ein hoher Frage- und Informationsbedarf der Patient:innen (52,9 %), unzureichende digitale Unterstützungssysteme (47,6 %) sowie eine hohe medizinische Komplexität der Patient:innen (42,5 %). Darüber hinaus nannten 26,1 % den Zeitaufwand für die Korrektur von Fehlinformationen und 23,1 % die verständliche Erklärung medizinischer Sachverhalte als Gründe für Zeitdruck (Abb. 2).

### Nutzung von KI-Tools

Die Mehrheit der Teilnehmenden gab an, KI-Tools privat und beruflich sowie rein privat zu nutzen (ca. 74 %). Die ausschließlich berufliche Nutzung wurde seltener angegeben (ca. 4 %). Etwa 19 % gaben an bislang keine KI-Anwendungen genutzt zu haben.

**Abb. 3:**
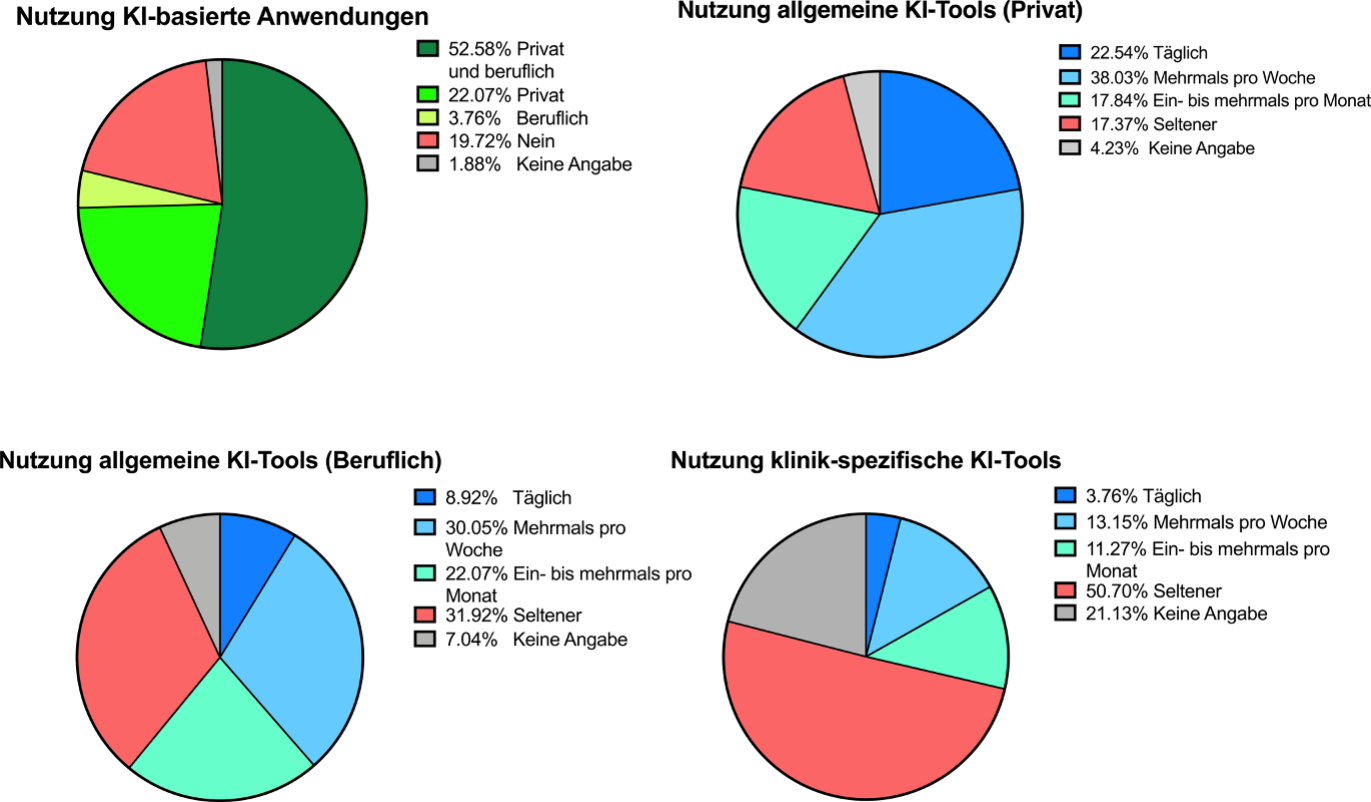
KI-Nutzung (künstliche Intelligenz) nach Kontext und Häufigkeit (*n* = 433). Allgemeine KI-Tools: Frei verfügbare Anwendungen ohne primär medizinische Zweckentwicklung; klinikspezifische KI-Tools: für klinische bzw. medizinische Anwendungen entwickelte Systeme

Allgemein zugängliche KI-Tools wurden häufiger verwendet als klinik- bzw. medizinisch spezifische KI-Anwendungen. Im privaten Kontext nutzten rund 20 % solche Tools häufig und weitere 36 % gelegentlich. Im beruflichen Kontext lagen die entsprechenden Anteile niedriger; zugleich berichtete rund ein Drittel der Befragten keine berufliche Nutzung allgemeiner KI-Tools. Klinik- bzw. medizinisch spezifische KI-Anwendungen wurden am seltensten eingesetzt. Etwa 51 % gaben keine Nutzung an, weitere rund 23 % waren hierzu unsicher bzw. machten keine Angabe (Abb. 3).

### Aufgabenbezogener Einsatz von KI im klinischen Alltag

Zur differenzierten Erfassung der KI-Nutzung wurde zwischen verschiedenen klinischen Anwendungsbereichen unterschieden. Für jeden Anwendungsfall wurde erhoben, ob die Teilnehmenden bereits allgemein zugängliche KI-Tools oder klinik- bzw. medizinisch spezifische KI-Anwendungen einsetzen. Ergänzend wurde mit der Antwortoption „Nein, aber ich würde gerne“ der potenzielle Bedarf an KI-Unterstützung erfasst.

Am häufigsten wurde KI für Informationssuche bzw. Recherche genutzt (59 %; 13 % klinikspezifisch, 46 % allgemein), gefolgt von Übersetzungen (52 %; 8 % klinikspezifisch, 44 % allgemein) und dem Erstellen oder Verbessern von Texten (46 %; 10 % klinikspezifisch, 36 % allgemein). Auch beim Verfassen von E‑Mails wurde KI von 27 % der Befragten eingesetzt.

Bei stärker klinisch geprägten Aufgaben fiel die aktuelle Nutzung geringer aus. KI wurde von 24 % zur Erstellung von Medikationsplänen bzw. zum Erkennen von Arzneimittelinteraktionen, von 23 % für medizinische Zweitmeinungen in komplexen Fällen und von 21 % für die Befund- und Arztbrieferstellung genutzt. Für Terminvereinbarung und Planung, Telefonie sowie die Analyse bildgebender Verfahren lagen die Nutzungsanteile jeweils nur zwischen 11 % und 13 %.

**Abb. 4:**
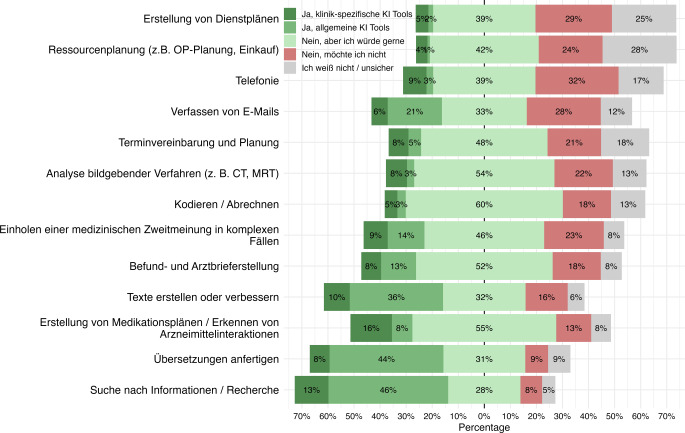
KI-Nutzung (künstliche Intelligenz) nach Anwendungsfällen im urologischen Alltag (*n* = 433). Allgemeine KI-Tools: Frei verfügbare Anwendungen ohne primär medizinische Zweckentwicklung; klinikspezifische KI-Tools: für klinische bzw. medizinische Anwendungen entwickelte Systeme

Besonders häufig wurde ein Nutzungswunsch für Kodieren und Abrechnen (60 %), Medikationspläne bzw. Arzneimittelinteraktionen (55 %), die Analyse bildgebender Verfahren (54 %), Befund- und Arztbrieferstellung (52 %), Terminvereinbarung und Planung (48 %) sowie medizinische Zweitmeinungen in komplexen Fällen (46 %) angegeben. Diese Ergebnisse weisen auf einen ungedeckten Bedarf für KI-Unterstützung insbesondere bei dokumentations-, organisations- und entscheidungsunterstützenden Tätigkeiten hin (Abb. 4).

### Voraussetzungen für besseres Vertrauen in KI-gestützte Systeme

Als häufigste Voraussetzung für ein besseres Vertrauen in KI-Systeme wurde eine hohe und überprüfbare Qualität der KI-Ausgaben genannt (82,4 %). 72,1 % forderten die Nutzung aktueller und evidenzbasierter Datenquellen, 70,0 % klare gesetzliche und regulatorische Rahmenbedingungen.

**Abb. 5:**
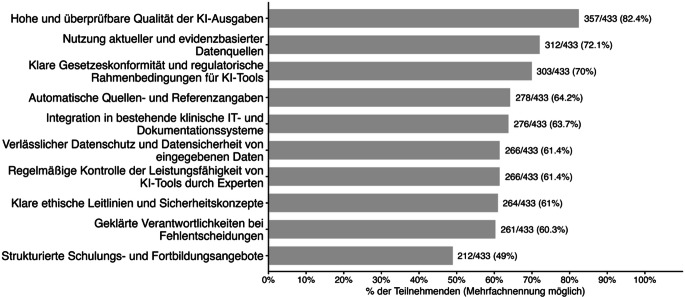
Angegebene Voraussetzungen für Vertrauen in KI-Systeme (künstliche Intelligenz, Mehrfachnennungen; *n* = 433)

Weitere Voraussetzungen waren automatische Quellen- und Referenzangaben (64,2 %), die Integration in bestehende klinische IT- und Dokumentationssysteme (63,7 %), verlässlicher Datenschutz und Datensicherheit der eingegebenen Daten (61,4 %) sowie eine regelmäßige Kontrolle der Leistungsfähigkeit von KI-Tools durch Expert:innen (61,4 %). Klare ethische Leitlinien und Sicherheitskonzepte wurden von 61,0 % genannt, geklärte Verantwortlichkeiten bei Fehlentscheidungen von 60,3 %. Strukturierte Schulungs- und Fortbildungsangebote hielten 49,0 % der Befragten für notwendig (Abb. 5).

### Erwartungen an zukünftige Auswirkungen

Am häufigsten erwarteten die Befragten eine Zeitersparnis im Arbeitsalltag (75,1 %). Darüber hinaus gingen 47,6 % von einer stärkeren Personalisierung KI-gestützter Behandlungspläne und 43,4 % von verbesserten Behandlungsergebnissen aus.

**Abb. 6:**
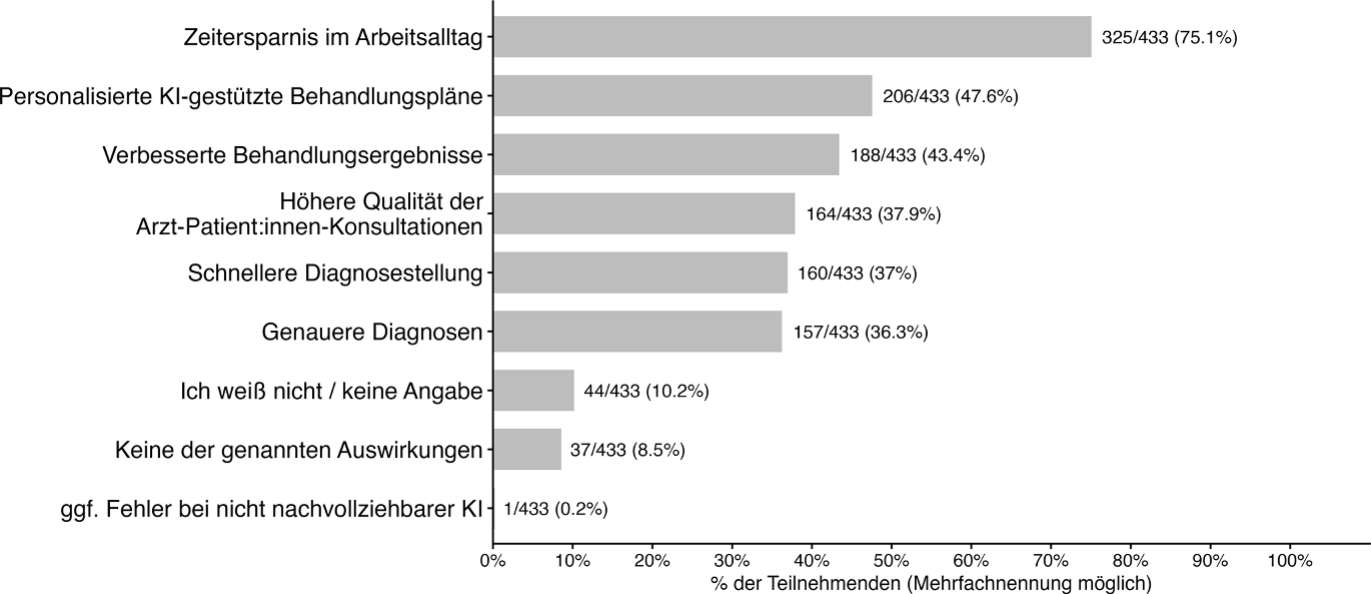
Angegebene erwartete Auswirkungen von künstlicher Intelligenz (KI) auf die Versorgung (Mehrfachnennungen; *n* = 433)

Weitere erwartete Effekte betrafen eine höhere Qualität der Arzt-Patienten-Konsultationen (37,9 %), eine schnellere Diagnosestellung (37,0 %) sowie präzisere Diagnosen (36,3 %). Insgesamt 10,2 % der Befragten gaben an, die zukünftigen Auswirkungen von KI nicht einschätzen zu können; 8,5 % erwarteten keine der genannten Effekte (Abb. 6).

## Diskussion

Das KI-Barometer zielt darauf ab, Arbeitsbelastung, aktuelle KI-Nutzung sowie Erwartungen im urologischen Versorgungsalltag zu erheben. Die Ergebnisse sprechen für eine wahrgenommene Arbeitsverdichtung bei den Befragten, als Hauptgründe hierfür geben die Teilnehmer administrative und dokumentationsbezogene Aufgaben an. Zugleich wird KI von einem Großteil der Befragten bereits im privaten und/oder beruflichen Kontext verwendet. Dabei kommen frei verfügbare allgemeine KI-Tools häufiger zum Einsatz als klinikspezifische Systeme. Die Befragten zeigen zudem auf, für welche klinischen Aufgaben KI als Entlastungsinstrument häufiger gewünscht wird. Hinweise auf einen ungedeckten Bedarf bestehen insbesondere bei Kodierung, Medikationsmanagement, Bildgebungsanalyse sowie Befund- und Arztbrieferstellung.

Bezogen auf die Arbeitsverdichtung und Dokumentationsaufgaben im medizinischen Bereich, decken sich die Ergebnisse des KI-Barometers mit der internationalen Literatur. Im ambulanten Setting wird im Durchschnitt 49,2 % der Arbeitszeit für Dokumentation und 27 % für direkte Patienteninteraktion aufgewendet [17]. Dokumentationsaufgaben gelten zudem als etablierter Risikofaktor für das Auftreten von Burnout-Symptomen [18, 19]. Der Wunsch nach KI-Unterstützung bei klinischen Aufgaben wie Kodierung oder Befund- und Arztbrieferstellung lässt sich vor diesem Hintergrund als Reaktion auf einen hohen Dokumentationsdruck interpretieren. Im KI-Barometer wurden diese Anwendungsfelder häufig als gewünscht, aber bislang nicht verfügbar beschrieben. Perspektivisch kann KI daher insbesondere bei der Dokumentation effektiv zur Entlastung von medizinischem Personal beitragen, z. B. durch Ambient-AI-Scribes. Unter dem Begriff Ambient-AI-Scribes werden KI-Systeme zusammengefasst, die aus dem Zusammenspiel von Spracherkennung und Sprachverarbeitung (z. B. mittels LLM) eine automatisierte Zusammenfassung von Gesprächen generieren können. Studien berichten über eine Reduktion der Dokumentationszeit von 8,5–20,4 % unter Zuhilfenahme von Ambient AI [20–22]. Gleichzeitig wurden Verbesserungen von Arbeitsbelastung und Burnout-Symptomen berichtet [22].

Auffällig ist zudem, dass sich frei verfügbare KI-Anwendungen schneller im Arbeitsalltag etablieren als klinikspezifische oder zugelassene KI-Systeme. Der Elsevier-Report zeigt, dass etwa die Hälfte der Befragten KI beruflich nutzt und 97 % dieser Gruppe dabei auf allgemeine Tools zurückgreifen [5]. Auch die ChatGPT-Studie 2025 des TÜV-Verbands weist mit 65 % aktiver Nutzung auf eine breite gesellschaftliche Verankerung hin [16]. In den USA nutzen mehr als die Hälfte der Erwachsenen regelmäßig KI-gestützte Chatbots und etwa zwei Drittel der Prompts entfallen dabei auf alltägliche Informationsrecherchen, bei denen Chatbots wie klassische Suchmaschinen (z. B. Google) verwendet werden [23]. Ein ähnliches Bild zeigt das aktuelle KI-Barometer. Frei zugängliche Anwendungen werden häufiger verwendet als medizinisch zugelassene Anwendungen. KI kommt dabei mutmaßlich v. a. dort zum Einsatz, wo keine direkten Patientendaten erforderlich sind oder die Aufgaben stark text- und wissensbasiert sind, etwa bei Recherche, Übersetzung oder Textentwürfen.

Die in dieser Studie angegebenen Voraussetzungen für Vertrauen in KI, insbesondere Qualität, evidenzbasierte Datenquellen und regulatorische Rahmenbedingungen, entsprechen bekannten Implementierungsbarrieren aus der Literatur. Eine Übersichtsarbeit identifizierte 19 Barrieren auf 3 Ebenen: technische und algorithmische Faktoren wie mangelnde Erklärbarkeit und fehlende Validierung, Stakeholder-bezogene Faktoren wie Vertrauensdefizite und Workflow-Probleme sowie gesellschaftliche Faktoren wie Datenschutz und Haftungsfragen [24]. Weitere Arbeiten betonen zusätzlich Interoperabilität, Datenqualität, Workflow-Integration und „change management“ als zentrale Herausforderungen [25–27]. Gerade der Aufwand für Datenmanagement und organisatorische Veränderungsprozesse wird von Institutionen häufig unterschätzt, was Implementierungen erheblich erschweren oder scheitern lassen kann [27]. Zukünftige Forschung sollte kontrollierte Implementierungsstudien mit Outcome-Messungen durchführen, um kausale Zusammenhänge zu etablieren [28, 29]. Die aktuelle Literatur und vorliegenden Ergebnisse des KI-Barometers legen nahe, dass erfolgreiche KI-Implementierung multidimensionale Interventionen erfordert: technische Lösungen (Interoperabilität, Datenqualität), organisatorische Maßnahmen (Training, Workflow-Integration) und regulatorische Klarheit [27–30].

### Limitationen

Limitationen der Studie ergeben sich v. a. aus dem explorativen Querschnittdesign und der nicht repräsentativen Stichprobe. Aufgrund der Rekrutierung über den DGU-Verteiler und der begrenzten Rücklaufquote ist ein Selektionsbias, insbesondere zugunsten KI-affiner Teilnehmender, möglich. Zudem beruhen die Ergebnisse auf Selbstangaben und können daher durch subjektive Wahrnehmung und Antwortverhalten beeinflusst sein. Der eigenentwickelte Fragebogen wurde nicht formal psychometrisch validiert; entsprechend wurden keine Reliabilitäts‑, Validitäts- oder Skalenanalysen durchgeführt. Die Auswertung erfolgte bewusst deskriptiv, sodass keine kausalen Aussagen oder belastbaren Subgruppenvergleiche möglich sind. Zudem wurden KI-Anwendungsfelder aufgabenbezogen erfasst, ohne systematische Differenzierung nach klinischer Tragweite, Risikoprofil oder regulatorischem Status. Die Ergebnisse sind daher primär explorativ und hypothesengenerierend zu interpretieren.

## Fazit

Die Ergebnisse der Befragung von 433 urologisch tätigen Teilnehmenden weisen auf eine wahrgenommene Arbeitsverdichtung hin, insbesondere durch administrative Aufgaben, Dokumentation und hohe Patient:innenzahlen. KI wird bereits relevant genutzt, v. a. in Form allgemein zugänglicher Tools und überwiegend für Recherche- und Textaufgaben. Zugleich zeigen sich Hinweise auf ungedeckten Bedarf bei administrativen und klinisch unterstützenden Tätigkeiten. Für eine breitere Implementierung erscheinen Transparenz, Qualitätssicherung und regulatorische Klarheit zentral; die zukünftigen Effekte von KI werden insgesamt überwiegend positiv eingeschätzt.

## Data Availability

Alle dieser Arbeit zugrunde liegenden Daten sind in diesem Artikel enthalten.
